# Tracking sero-molecular trends of swine brucellosis in Hawai‘i and the central Pacific

**DOI:** 10.3389/fpubh.2024.1440933

**Published:** 2024-09-04

**Authors:** Thi Hai Au La, Ian A. McMillan, Prashant Dahal, Andrew H. Burger, Mahdi Belcaid, Darrin M. Phelps, Samuel M. Goldstein, Vienna R. Brown, Michael H. Norris

**Affiliations:** ^1^Pathogen Analysis and Translational Health Group, School of Life Sciences, University of Hawai‘i at Mānoa, Honolulu, HI, United States; ^2^Department of Oceanography, School of Ocean and Earth Science and Technology, University of Hawai‘i at Mānoa, Honolulu, HI, United States; ^3^Department of Information and Computer Sciences, University of Hawai‘i at Mānoa, Honolulu, HI, United States; ^4^Wildlife Services, United States Department of Agriculture, Honolulu, HI, United States; ^5^U.S. Department of Agriculture, Animal and Plant Health Inspection Service, Wildlife Services, National Wildlife Disease Program, Honolulu, HI, United States; ^6^National Feral Swine Damage Management Program, United States Department of Agriculture, Fort Collins, CO, United States

**Keywords:** brucellosis, *Brucella*, feral swine, Hawai‘i, Asia-Pacific, zoonoses, molecular epidemiology, zoonotic disease

## Abstract

**Introduction:**

Brucellosis is a zoonotic disease of mammals caused by bacterial species of the *Brucella* genus. The reservoir for disease is typically mammals, with species of *Brucella* found infecting amphibians, bats, and marine mammals. *Brucella* spp. can pass directly to humans through contact with infected animals or their products. *Brucella* spp. can cause chronic debilitating infections in mammals, including humans, and is associated with spontaneous abortions in infected animals, causing reduced fecundity. In Hawai‘i, terrestrial species that could harbor *Brucella* spp. include swine, cattle, horses, and axis deer among others. The numerous feral swine in Hawai‘i are known to carry *Brucella suis*, with evidence supporting infections in cattle. *Brucella suis* also poses infection risk to humans, dogs, and potentially horses across the state.

**Methods:**

In this study, 3,274 feral swine serum samples collected from 5 of the 8 main islands over a 15-year span were analyzed for exposure to *B. suis*. Of the 558 watersheds in the state, 77 were sampled as part of this effort. Spatial analysis was used to identify watersheds of concern. MLVA and whole genome SNP analysis was used for molecular epidemiological analysis.

**Results:**

Statewide seropositivity rates were triple that of feral swine found in the conterminous United States. Smoothed positivity rates were highest on Maui, followed by O‘ahu, and the island of Hawai‘i. Island-by-island analysis found high brucellosis positivity levels associated with specific watersheds and agricultural areas. Local spatial autocorrelation identified hot spots on O‘ahu and Hawai‘i. MLVA analysis of available *B. suis* from Hawai‘i found molecular epidemiological connections with *B. suis* found in French Polynesia and the mainland US while differing from those in Tonga, Western Polynesia. Strains from Hawai‘i are phylogenetically closest to strains from the United States. MLVA and SNP analysis found *B. suis* strains from Hawai‘i fell into the genetic group that contains biovar 1 *B. suis*.

**Discussion:**

This work identified islands and watersheds of high brucellosis seropositivity in feral swine of Hawai‘i, highlighting the magnitude of the zoonotic risk. Introduction of strains in recent history is unlikely due to modern animal trade and disease control practices. Genomic analysis of strains in Hawai‘i and the Pacific area can provide hidden historical and local clues to brucellosis epidemiology in the state.

## Introduction

The zoonotic disease brucellosis is also known as Malta fever or undulant fever. The monikers are in reference to the geographic location where brucellosis was first identified and the common recurrent fevers observed in human patients (1). Brucellosis is a chronic syndromic infection commonly resulting in chronic infections that can lead to or co-occur with spontaneous abortions, fevers, arthritis, and infections of the male or female reproductive tracts (2–4). The slow progression of the infection is characterized by lethargy, malaise, general pain, and weight loss (5, 6). It is not usually fatal on its own but weakens the host, making them more susceptible to comorbidities (7). The disease in humans is caused by six species in the *Brucella* genus that are often host-adapted (8). *Brucella* spp. have host preferences but most virulent strains can infect multiple host species; observable as frequent spillover from one host to another (9–13). The Gram-negative *Brucella* bacteria are highly infectious and can be spread by aerosol contact with mucosal surfaces and contact with infected biological materials such as milk or aborted material (14).

Globally, the most important source for brucellosis transmission to humans is domesticated animals. Where possible, infections in agricultural animals are monitored by testing milk or serum for antibodies to the bacterial lipopolysaccharide. In areas with developed veterinary disease programs, brucellosis-positive animals are culled to prevent disease transmission, reduce economic losses and protect public health. In many places, including the US and Europe, wildlife such as wild boar, can harbor *Brucella* infections that spread to domesticated animals and humans (15–17). In the geographically isolated Hawaiian archipelago, terrestrial wildlife species are more limited than continental areas. Starting with the arrival of Polynesian travelers hundreds of years ago, humans have moved food source animals to the islands. With the arrival of European colonizers, the Eurasian wild boar was introduced (18). Feral swine are an important cultural and sport-hunting food source in Hawai‘i (19, 20), though the populations are uncontrolled. In recent times, rising nuisance behaviors that destroy property and carriage of disease has increased attention on controlling the feral swine population across the United States (US) and Hawai‘i. The diversity of the terrain in Hawai‘i coupled with ample tree cover make eradication challenging, necessitating targeted control efforts.

The feral swine in Hawai‘i are known to carry brucellosis and presumably spread it to disease-free domestic stock, including swine and cattle (21). Reducing the population of disease carrying animals would limit threats to agricultural and public health. This work analyzed 3,274 feral swine over 15 years across the state of Hawai‘i. The samples were tested for seroreactivity using a two-stage testing procedure including the *Brucella* acidified plate antigen (BAPA) test and *Brucella suis* fluorescent polarization assay (FPA) (22–24). Positivity rates were analyzed by year and spatially. The spatial analysis identified islands of highest brucellosis seropositivity during the 15-year period. Rates were smoothed to account for uneven sampling across the state and then by island. Smoothed rates were determined at the watershed level for each island and local spatial autocorrelation analysis (LISA) allowed identification of O‘ahu and Hawai‘i watersheds with the highest positivity rates. Genetic analysis of global *B. suis* strains multi-locus variable number tandem repeat array (MLVA) profiles was used to identify ecogeogenetic linkages of three *B. suis* cattle isolates from Hawai‘i to regional Polynesian strains and others from around the world. Whole genome SNP analysis was used to confirm MLVA findings with available whole genome data. This study is the first to look at long term brucellosis trends in Hawai‘i. The high spatial resolution achieved at the watershed level will inform policy makers and stakeholders in efficient deployment of animal control resources while the genetic analysis provides clues to the placement of *B. suis* from Hawai‘i in the global brucellosis genetic landscape.

## Materials and methods

### Swine sampling

Feral swine were sampled across the state of Hawai‘i by the United States Department of Agriculture Animal and Plant Health Inspection Service Wildlife Services National Feral Swine Damage Management Program. USDA personnel responded to landowner animal trappings on O‘ahu or in organized trapping programs in response to animal nuisance activities on O‘ahu and neighboring islands. Anesthesia was not used on any feral swine in the study and euthanasia of feral swine was conducted by gunshot to the head in accordance with the American Veterinary Medical Association (AVMA) Guidelines for the Euthanasia of Animals (25), whereby a projectile fired from a firearm enters the brain, causing instant loss of consciousness. While GPS coordinates of individual animal trappings were recorded, animal location was reported to the watershed level to protect identification of landowners. Feral swine capture and euthanization were done so with the consent of property owners/managers through a mutually-signed Work Initiation Document, wherein the owner/manager agreed to allow Wildlife Services personnel on property to enumerate the target species and perform euthanasia. Blood was collected from swine carcasses by post-mortem heart puncture and the serum was separated. Serum samples were shipped to the USDA APHIS Veterinary Services Federal Brucellosis Laboratory in Frankfort, Kentucky and tested by the BAPA test followed by the FPA for confirmation of *Brucella* specific antibodies according to the manufacturer's guidelines (Ellie Lab LLC; Germantown, Wisconsin, USA) and the USDA Standard Operating Procedures for Submission and Testing of Brucellosis Serological Specimens guidance document. Year, watershed, and USDA brucellosis positivity results for feral swine tested are listed in Supplementary Table S1.

### Seropositivity rate analysis

Seropositive animals for each island tested were divided by total number of animals tested over indicated periods for raw rate determination. Number of cases and seropositives were plotted along with raw rate averages using the GraphPad Prism software. Raw rates across the island were visualized in QGIS 3.23 Lima (26). Empirical Bayesian smoothing (EBS) in GeoDa (27–30) was used because of the incontiguous nature of islands and to account for watersheds where few samples were collected. Unsampled watersheds were not included in the analysis. Smoothed rates were visualized across the state in QGIS. Islands with contiguous watershed sampling and positive samples were analyzed individually by spatial Bayesian smoothing (SBS) (31) using the first-order queen's contiguity matrix produced for each in GeoDa to smooth the random spatial sampling effects to the means (32, 33). Maui was excluded from SBS analysis due to sampling of only 3 non-contiguous watersheds. More complete sampling coverage of Honolulu and Hawai‘i counties meant many watersheds sampled on the islands of O‘ahu and Hawai‘i were contiguous and could benefit from SBS. Unless otherwise indicated place names refer to the island not the county throughout this manuscript. Smoothing is a method for dealing with rate instability, especially when sampled areas are not equally sampled or their sizes differ considerably, as the size of a watershed could also affect observed rates. Another smoothing assumption is that the real rates in contiguous watersheds are likely reflective of each other since they are spatially linked. Our reasoning for choosing EBS or SBS based on contiguity of neighboring watersheds is described above. Simply put, the smoothed rates of each watershed are moved toward the overall spatial unit average, moving most probable outliers (high and low) toward that mean and reducing observed rate instability due to samples biases.

### Local spatial autocorrelation analysis (LISA)

To test for hotspots, local spatial autocorrelation analysis (LISA) was performed separately for Honolulu County (island of O‘ahu) and Hawai‘i County by calculating the univariate Local Moran‘s I statistics with the GeoDa default 999 permutations using the spatially smoothed seropositivity rates for each in GeoDa. Clusters identified with p ≤ 0.05 were visualized on a map of the respective island counties to indicate watershed areas of most and least concern in high-high, high-low, low-low, or low-high clusters if significant.

### MLVA-11 and MLVA-16 analysis

MLVA-11 and MLVA-16 profiles for all *Brucella* in the *Brucella* MLVAbank Microbes Genotyping database (34) were extracted, totaling 20,982 MLVA-11 strain profiles. Incomplete profiles were not included in the anlaysis. MLVA-16 profiles of *B. suis* were extracted MLVAbank and numbered 695 strain profiles. The MLVA profiles deposited in MLVAbank provide a historical snapshot of MLVA profiles when next sequencing did not exist or was not as accessible as it is today. Raw sequence reads of seven *B. suis* strains from Tonga (Western Polynesia) and three strain from Hawai‘i (Northern Polynesia) were downloaded from NCBI (35). The genomes were assembled and annotated using the Nextflow core (36) bacass bacterial genome assembly pipeline (37) run on the University of Hawai‘i at Mānoa high performance Koa computer. The MLVA profiles and metadata of assembled genomes from Tonga and Hawai‘i were obtained using MLVAfinder python code (https://github.com/i2bc/MLVA_finder) and added to the *B. suis* MLVA profiles downloaded from MLVAbank. MLVA profiles were used to build phylogenetic trees on a local instance of grapetree and visualized with relevant metadata (38). Neighbor joining trees were constructed using the RapidNJ (39) in grapetree. MLVA-16 neighbor joining trees were created with profiles of biovar 1 *B. suis* to analyze the geogenetic relationships of *B. suis* biovar 1 in the Pacific Region. MLVA-16 loci were weighted to account for unequal variability among the MLVA-16 loci (40).

### Whole genome SNP analysis

Sequence read archive files for 258 *B. suis* genomes, including seven strains from Tonga and three from Hawai‘i (Supplementary Table S2), were extracted from the European Nucleotide Archive and assembled with the Nextflow core bacass bacterial genome assembly pipeline as described above on the University of Hawai‘i Koa high performance computer cluster. Assembly statistics for the whole genomes are provided as Supplementary Table S3. SNP analysis was performed using the PhAME package (41). Briefly, NUCmer is used to perform pairwise alignments between all genomes. Gaps, repeats, and indels were identified and removed to generate a “core” genome. The contigs are then compared to a reference, *B. suis* 1330 (GCF_000223195.1) in this case, using NUCMer. SNP coordinates in the “core” genome were used to generate genome-wide core alignments for the maximum likelihood SNP trees. SNP trees were built and bootstrapped using RAxML and the resulting trees visualized in iTOL (42).

## Results

**Figure 1:**
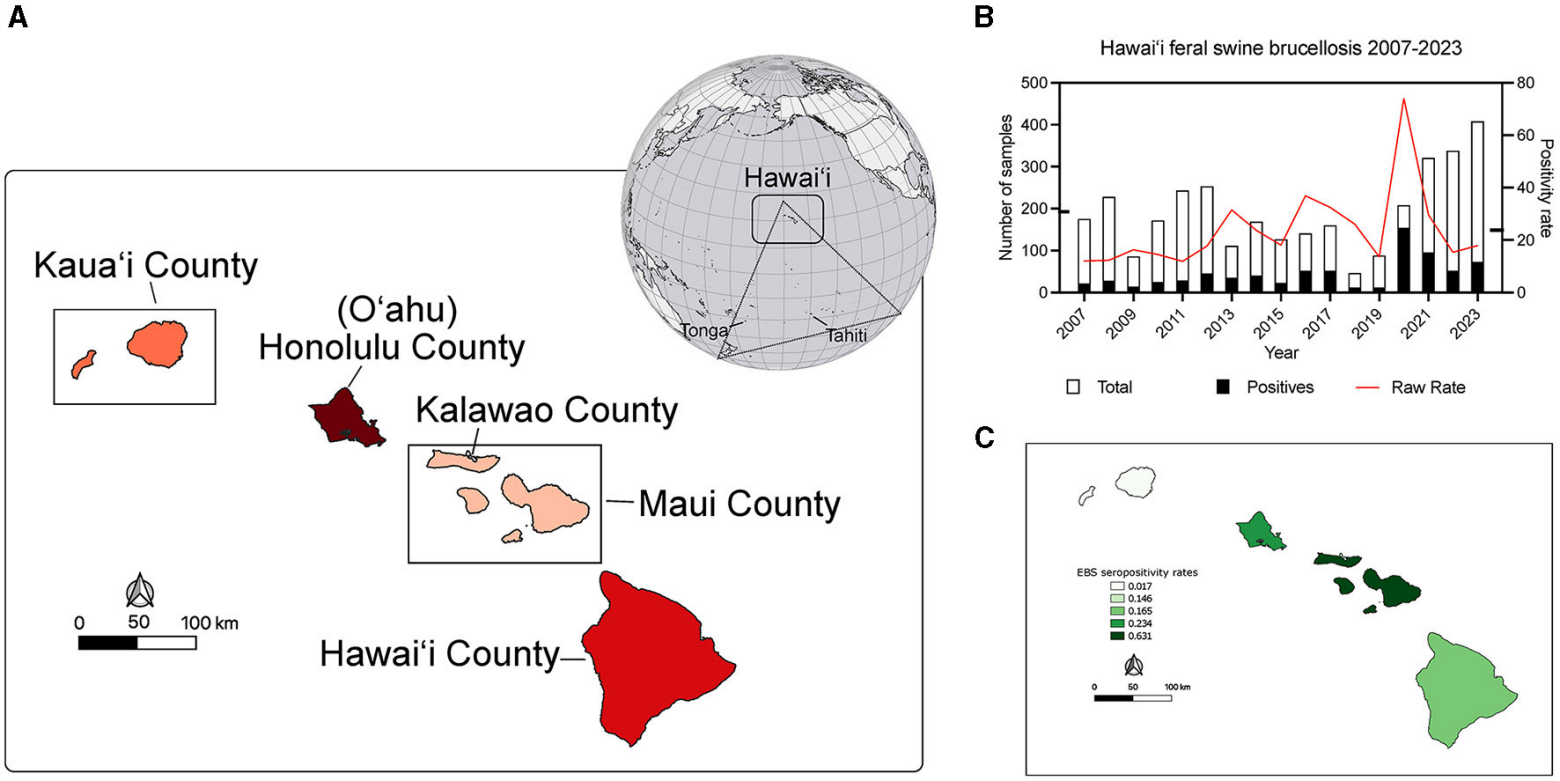
Location of Hawai‘i within Polynesia and brucellosis seropositivity rates in feral swine during the study period. **(A)** The globe shows the location of Polynesia in the Pacific Basin. Simplistically Polynesia can be thought of as a >300,000 sq km (>117,000 sq mi) triangular shaped region encompassing numerous islands in the Pacific Ocean. At the southwestern vertex of the triangle is New Zealand, the southeastern is Rapa Nui (Easter Island), and at the northern vertex are the islands of Hawai‘i. The rectangle on the globe indicates the location of Hawai‘i in Polynesia and the map shows the magnified view of the islands. The administrative county units and island names are indicated. The islands are shaded red according to the number of swine serum samples analyzed in this study (the darker the red the more samples) and coincide with the details in Table 1. **(B)** The number of serum samples (left y-axis) obtained each year from 2007 to 2023 (x-axis) are indicated in white vertical bars with the number of brucella FPA seropositive samples overlain in black. The raw yearly seropositivity rate is indicated by the red line (right y-axis). Notches on the y-axes indicate respective averages across the study period. **(C)** A map of the EBS rates of brucellosis feral swine seropositivity at the county level. The darker islands have higher EBS smoothed county seropositivity rates.

The majority of the feral swine samples (2,558/3,274) across the 15 years of study were from Honolulu County which is the island of O‘ahu (Table 1, Figure 1). O‘ahu is the most populated island in the state with 989,408 people of the total state population of 1,435,138 as of July 2023 according to the US Census Bureau (43). The county and island of Hawai‘i had the next highest number of samples at 329. The county of Kaua‘i includes the islands Ni‘ihau and Kaua‘i. All 212 Kaua‘i County samples were from the island of Kaua‘i. Maui County is composed of the islands of Maui, most of the island of Moloka‘i (except for the Kalaupapa area), Lana‘i, and Kaho‘olawe. The 165 samples from Maui were entirely from the island of Maui. Ten samples were from Kalawao County containing Kalaupapa on the north shore of Moloka‘i. Lana‘i is privately owned and Kaho‘olawe is uninhabited.

**Table 1:** Summary of sample locations.

**County**	**Islands**	**Islands sampled**	**# of samples**
Honolulu	O‘ahu	O‘ahu	2,558
Hawai‘i	Hawai‘i	Hawai‘i	329
Kaua‘i	Kaua‘i, Ni‘ihau	Kaua‘i	212
Maui	Maui, Moloka‘i, Lana‘i, Kaho‘olawe	Maui, Moloka‘i	165
Kalawao	Part of Moloka‘i	Part of Moloka‘i	10
Total samples			3,274

On average there were 192.6 (±97.30) samples collected every year and 44.82 (±35.86) were positive each year of the study as determined by the two stage BAPA and FPA brucellosis testing (Figure 1B). The average annual raw positivity rate is 23.77% (±15.21). Annual sampling minima was 46 in 2018 and a maxima of 408 in 2023 with an average of 193.69 samples each year. The minimum brucellosis seropositivity rate in feral swine occurred in 2011 at 11.93% of 243 swine sampled while the maximum occurred in 2020 at 74.04% of 208 swine sampled (Figure 1B). Of the sampled islands there were no positive samples from Kaua‘i or Moloka‘i.

**Figure 2:**
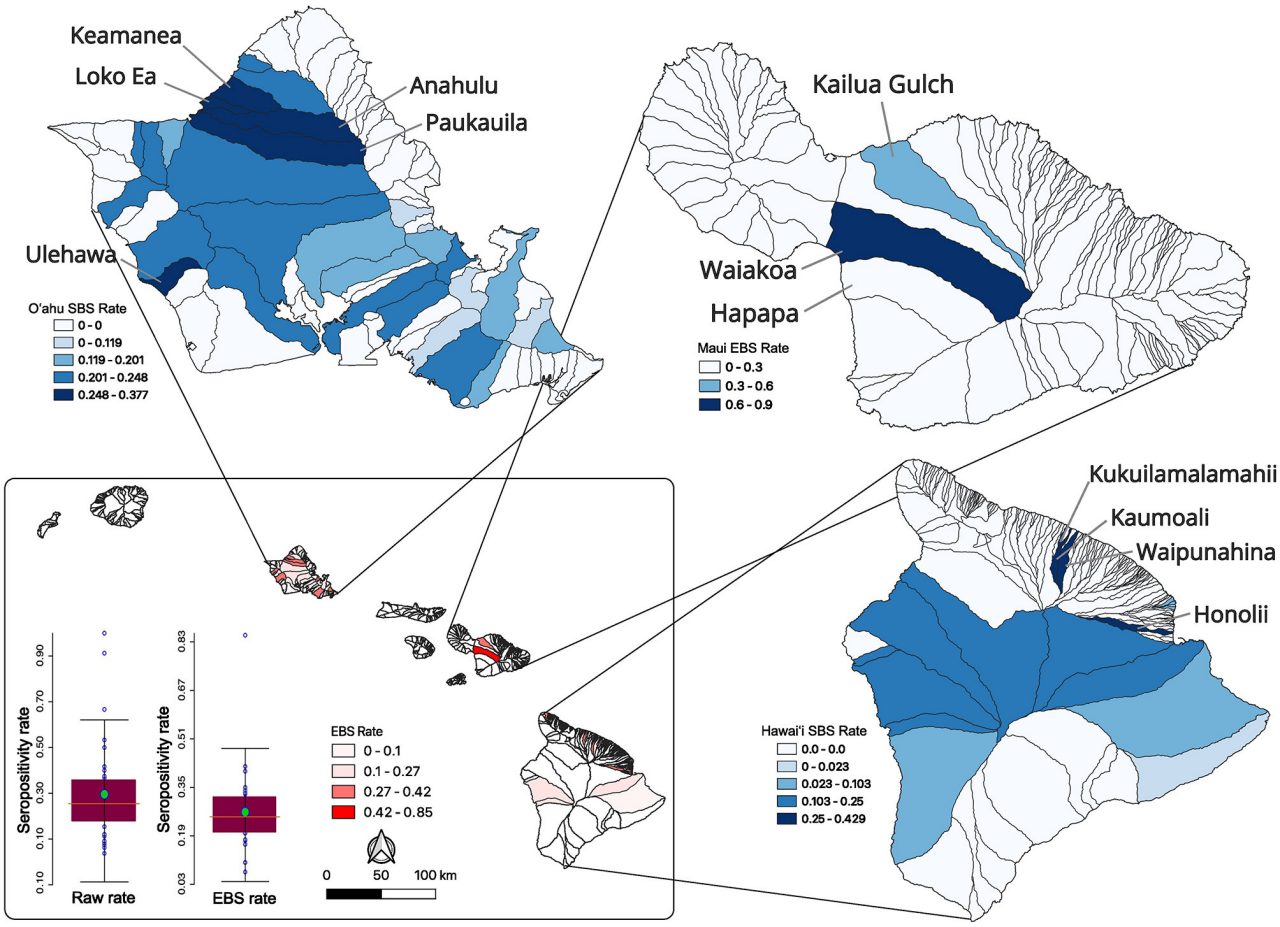
Feral swine serum sampling and seropositivity rates across Hawai‘i watersheds. Raw watershed seropositivity rates (bar graph on left; lower inset) were smoothed by statewide EBS (bar graph on right; lower inset) which reduced the mean and standard deviation of the rates across the state. The statewide smoothed EBS rates are shown in the inset map with darker watersheds having higher brucellosis swine seropositivity rates. Smoothing of data from individual islands is shown starting with SBS rates for O‘ahu **(upper left)** then moving clockwise to Maui EBS rates and then Hawai‘i SBS rates on the bottom right map. In all cases, the darker the fill the higher the smoothed seroprevalence for that watershed. High seroprevalence watersheds are labeled.

Low sample size or collection bias could impact the observed rates. In an attempt to account for such sampling biases, rates were smoothed at the county level using empirical Bayesian smoothing in GeoDa (Figure 1C). No samples were positive in Kaua‘i County however the smoothing increased the rate toward the mean and was equal to 1.7%. The Kalawao County positivity rate was smoothed to 14.6% and Hawai‘i County was smoothed to 16.5%. The Honolulu County (O‘ahu) rate was smoothed to 23.4% and Maui County swine samples had a smoothed brucellosis seropositivity rate of 63.1%. County level divisions are large and the diverse ecosystems within an island are not captured, so data were analyzed at the watershed level. The watershed encompasses a mauka to makai (mountain to sea) piece of the island akin to a slice of pie. In total there are 558 watersheds across the eight major islands in the state of Hawai‘i, and 77 out of 558 were sampled as part of this project (Supplementary Figure S1). The island of O‘ahu was heavily sampled and swine from 33 of its 87 watersheds were analyzed. Five watersheds on Kaua‘i, two watersheds from Kalawao County on Moloka‘i, and three from the island of Maui were sampled. Thirty four of 166 watersheds on the island of Hawai‘i were sampled. Altogether, feral swine were sampled from watersheds representing 51.29% (3,301.87/6,437.22 sq mi) of the total land area of the eight major Hawaiian Islands. Watershed positivity rates were smoothed, reducing the mean positivity rate from 0.2941 to 0.2662 and reducing the standard deviation from 0.2131 to 0.1280 (Figure 2; inset lower left). A major outlier before and after smoothing was a watershed on the island of Maui. Since Maui, O‘ahu, and Hawai‘i are geographically isolated, each island was analyzed independently to increase smoothing effects on each island's brucellosis rates. Watershed rates for O‘ahu and Hawai‘i were spatially smoothed because of the contiguity of watersheds that were sampled. The small number of non-neighboring watersheds sampled on Maui precluded appropriate application of SBS so EBS was applied (Figure 2; individual island maps). The smoothed rates in sampled watersheds ranged from 0% to 37.68% on O‘ahu, 8.25% to 90.11% on Maui, and 0% to 42.86% on Hawai‘i. The watershed of highest seropositivity was Anahulu on O‘ahu, Waiakoa on Maui, and Honoli‘i on Hawai‘i.

**Figure 3:**
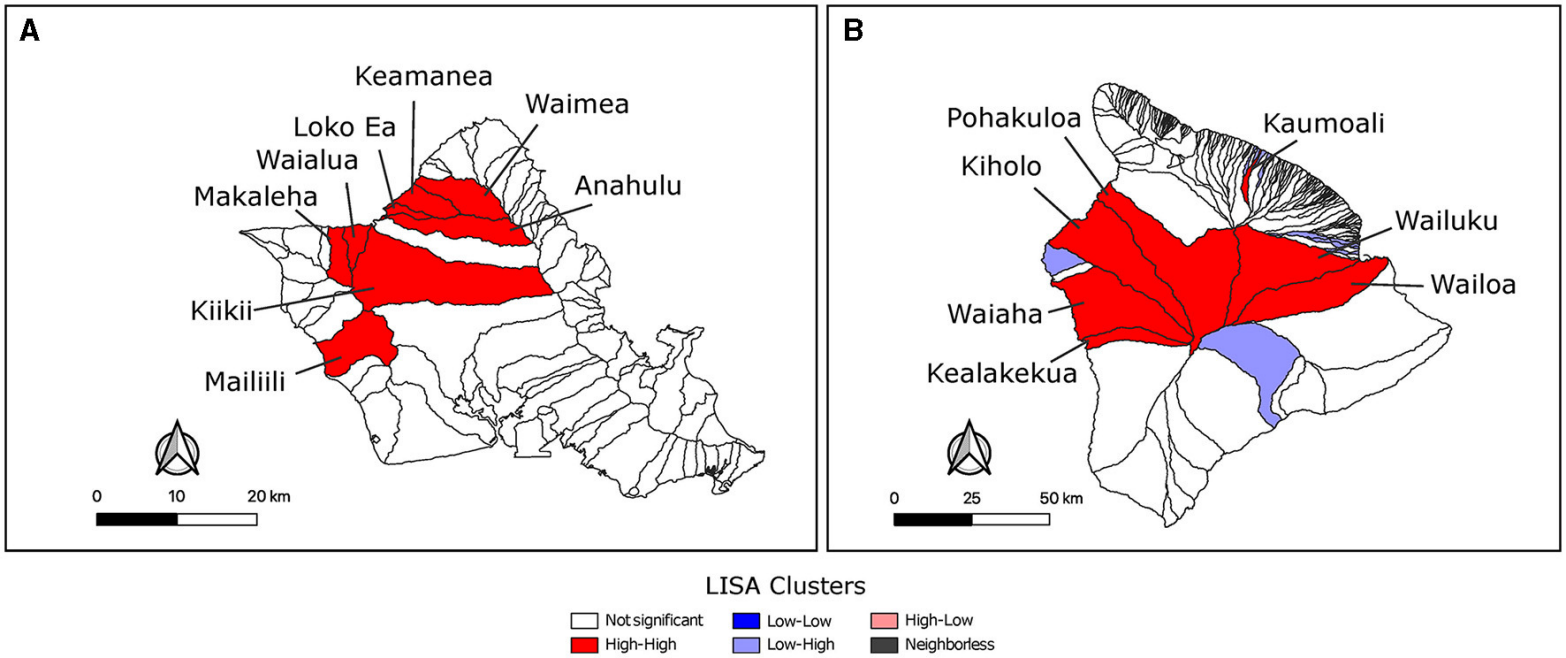
Identification of hot-spot clusters of feral swine brucellosis seropositivity. LISA analysis using Local Moran's I found significant clusters of brucellosis seropositivity on O‘ahu **(A)** and Hawai‘i **(B)**. Clusters with *p* values ≤ 0.05 are shown. Unfilled watersheds did not cluster significantly or were not sampled and left out of the analysis. Watersheds in high-high clusters are labeled with their names.

Feral swine from three watersheds were sampled on Maui where Waiakoa was a clear high prevalence outlier. The reduced number of sampled watersheds prevented a more thorough LISA analysis for hotspots on Maui. LISA hotspot analysis of the SBS rates was used to analyze O‘ahu and Hawai‘i for low and high seroprevalence areas of swine brucellosis (Figures 3A, B). There were only high-high clusters identified on O‘ahu (Figure 3A). High-high clusters are neighboring spatial units that each have statistically higher seropositivity than the mean and where the test for spatial randomness of those units has been rejected, indicating a cluster. One cluster on the north shore of the island consisted of Waimea, Keamanea, Loko Ea, and Anahulu watersheds and a second cluster nearby straddling the Waianae mountain range and central O‘ahu. The most statistically significant cluster was the Keamanea and Anahulu watersheds on the north shore. On the island of Hawai‘i, the major high-high cluster was located in watersheds between the two biggest volcanoes on the islands, Mauna Kea and Mauna Loa (Figure 3B). Statistically significant low-high clusters were identified in watersheds that abut this cluster.

**Figure 4:**
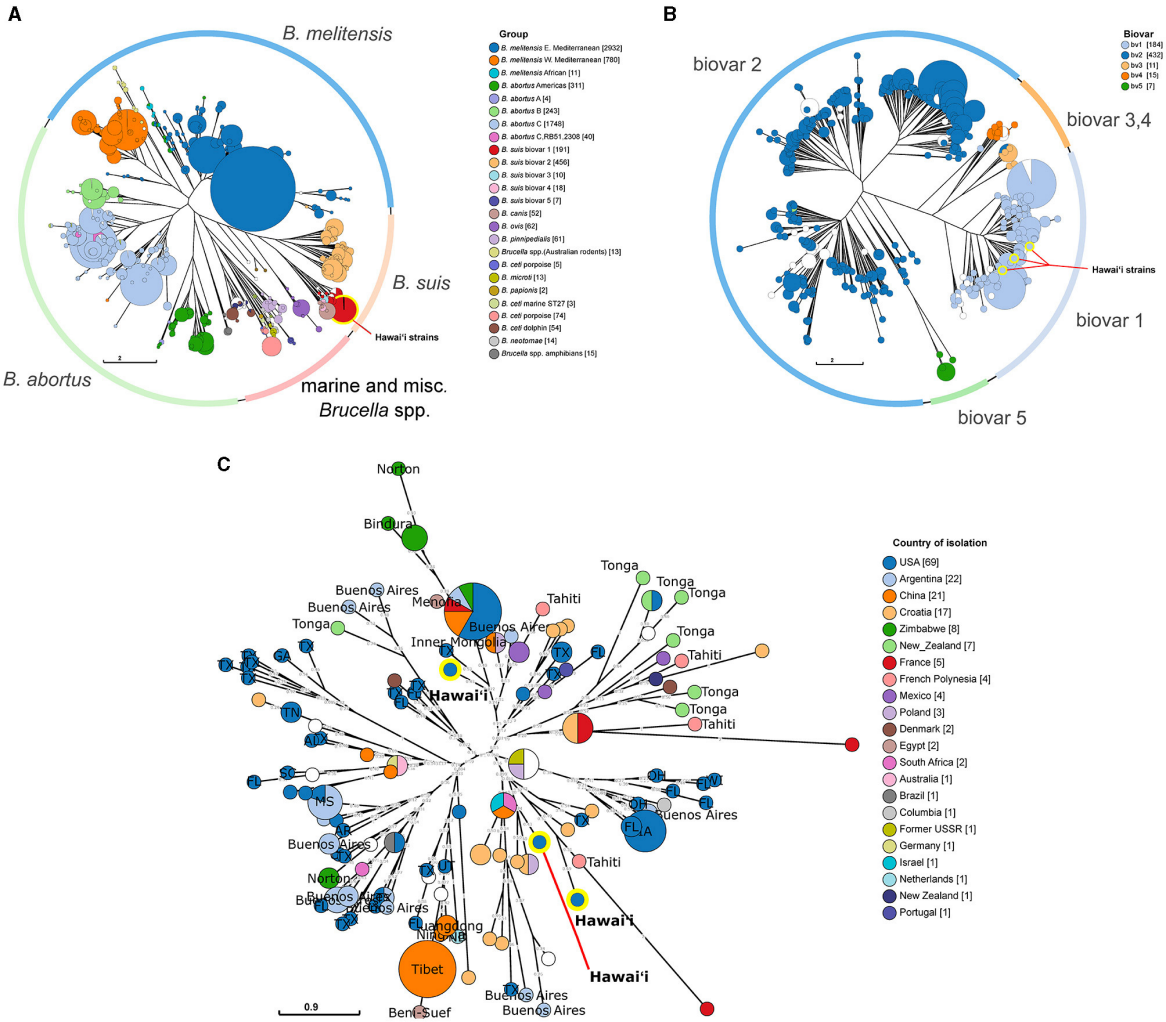
Phylogenetic relatedness of Hawai‘i *B. suis* to global *Brucella* isolates and regional *B.suis*. **(A)** Neighbor joining tree analysis of all available *Brucella* strains using the MLVA-11 panel. The outer ring indicates the location of specific *Brucella* spp. in the tree. Strains are clustered into MLVA-11 sequence types indicated by circles. Circles are colored according to the assigned *Brucella* group. **(B)** Neighbor joining tree analysis of all available *B. suis* strains using the MLVA-16 panel. The outer ring indicates the location of different *B. suis* biovars on the tree. As with the MLVA-11 tree in **(A)**, strains are clustered into MLVA-16 sequence types indicated by circles. Circles are colored according to biovar. The yellow highlighted *B. suis* circles indicate the three Hawai‘i *B. suis* biovar 1 strains. **(C)** A weighted neighbor joining tree of global *B. suis* biovar 1 strains. The *B. suis* biovar 1 were analyzed to find the closest related strains to the Hawai‘i *B. suis* isolates. The circles are colored according to the country of isolation and labeled with their state or subregion of isolation providing a geographic context to the sequence type clusters. US state two letter abbreviations are used. The location of the three Hawai‘i *B. suis* are highlighted in yellow on each tree. Numbers on the branches indicate the allelic differences between connected sequence types. Circle size in each tree corresponds to the number of strains within the indicated groups.

To provide clues to the spread of brucellosis to the islands of Hawai‘i, phylogenetic analyses of available genetic information regarding *Brucella* in Hawai‘i were performed with increasing genetic resolution. First, *in silico* MLVA-11 profiles were generated from assembled Hawai‘i *Brucella* whole genomes originally sequenced in (35), then the profiles were compared to historical *Brucella* strain MLVA-11 profiles from MLVAbank. MLVA-11 allowed clear species and geographic separation of the larger *Brucella* groups using neighbor joining tree phylogenetic analysis. The strains isolated from cattle in Hawai‘i fell into the *B. suis* group (Figure 4A; highlighted red circle). Increasing the MLVA loci utilized from MLVA-11 to MLVA-16 for the *B. suis* group found that the Hawai‘i cattle *B. suis* fell into the *B. suis* biovar 1 group (Figure 4B; highlighted blue circles). The *B. suis* biovar 1 group was analyzed further in a MLVA-16 weighted neighbor joining tree to find the closest non-Hawai‘i *B. suis* (Figure 4C). One strain from Hawai‘i was closely related to a strain of *B. suis* from Texas and was located across the central tree node from the other two Hawai‘i isolates. These two Hawai‘i *B. suis* strains were closely related to a strain from Tahiti, which is also in Polynesia ~4,200 km (~2,600 mi) south of Hawai‘i. Tahitian *B. suis* strain BCCN#02-28 collected in 2002 differs from Hawai‘i strain B00-0468 collected in 2000 by one repeat at Bruce16 MLVA-16 locus. The next closest Hawai‘i strain is B11-0525 and differs from *B. suis* B00-0468 by two repeats at Bruce04, two repeats at Bruce09, and one repeat at the Bruce16 loci. The third Hawai‘i *B. suis* strain B93-0748 was isolated in 1993 and differs from the year 2000 Hawai‘i *B. suis* B00-0468 at the Bruce04, Bruce07, Bruce09, and Bruce16. The third strain, B93-0748, from the year 1993 was isolated the earliest and is closest to a *B. suis* isolated in Texas in 2000. B93-0748 Hawai‘i differs from B00-0729 Texas by two repeats at the Bruce09 locus. Other Polynesian *B. suis* isolates with MLVA-16 profiles were more distantly related to the Hawai‘i strains, including several isolates from Tonga in the southwestern corner of Polynesia and additional isolates from Tahiti. Many of the Tongan *B. suis* isolates clustered together with strains from Tahiti, New Zealand, and Mexico.

**Figure 5:**
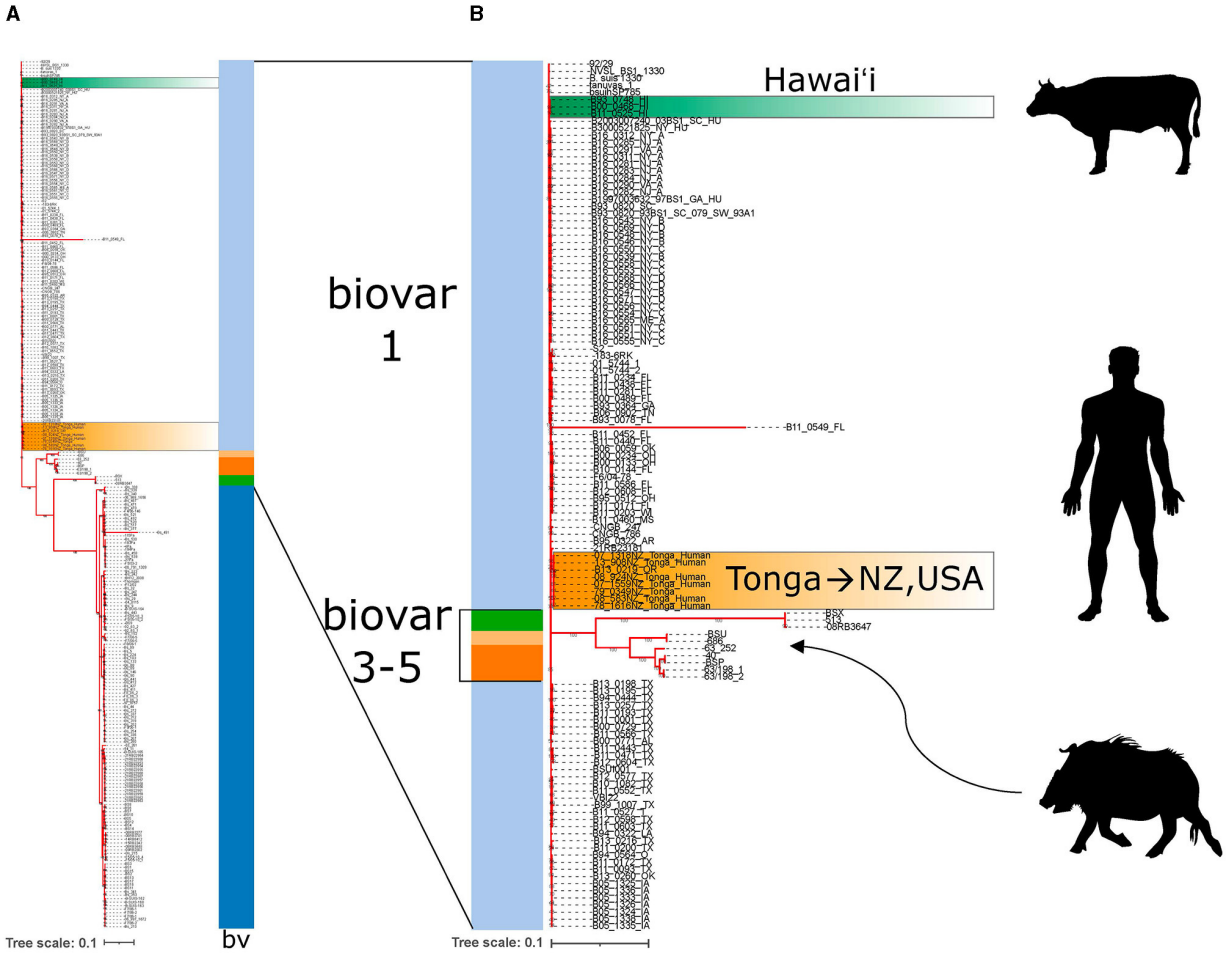
Whole genome SNP analysis of *Brucella suis* from Polynesia indicates multiple introductions of *B. suis*. **(A)** Whole genome SNP RAxML 100x bootstrapped phylogenetic tree of available *B. suis* genome sequences. Biovar data is indicated by the vertical bv strip where light blue = biovar 1, dark blue = biovar 2, dark orange = biovar 3, green = biovar 4, light orange = biovar 5. Dark green highlighted strains are the *B. suis* strains from Hawa‘i and orange highlighted strains are the *B. suis* strains from Tonga. **(B)** The biovar 1,3,4,5 whole genome sequenced *B. suis* strains are emphasized with silhouettes indicating the host species involved in *B. suis* strain isolations. Hawai‘i strains are from cattle. Tongan strains are from exposure in Tonga with subsequent cases diagnosed in New Zealand and Oregon, USA. The wild boar silhouette indicates where the Hawai‘i *B. suis* biovar 3 wild boar isolate described in literature would presumably be found on the tree if sequences were available.

There are many more strains of MLVA-typed *B. suis* than have been whole genome sequenced. Even then, whole genome sequencing can provide additional high resolution genomic context to support findings from MLVA comparisons. Strains from the United States make up the bulk of genome sequenced isolates. Whole genome reads of the three isolates from Hawai‘i were publicly available and included in whole genome SNP analysis with a total of 257 *B. suis* genomes. Isolates clearly partitioned into biovar categories as indicated by the color strip (Figure 5A). The Hawai‘i *B. suis* clustered with the biovar 1 isolates as found in the MLVA-16 trees. A closer look at the biovar 1, 3, 4 and 5 isolates found the Hawai‘i *B. suis* genomes were phylogenetically closer on the tree to other biovar 1 isolates from South Carolina and New York states on the US east coast. A strain of unknown origin named bsuihSP785-sc-2150388 is predicted to be closely related. All three whole genome sequenced Hawai‘i isolates neighbor each other on the tree with no SNPs in the core genome identified among these strains (Figure 5B). Altogether, the *B*. suis biovar 1 have low SNP diversity in the conserved core genome used in this analysis. In agreement with the MLVA-16 tree in Figure 4C, the Tongan isolates involved in exported cases of brucellosis in humans are distantly related to the three cattle isolates from Hawai‘i. There were no genomic signatures coinciding to a previously described *B. suis* biovar 3 in a feral swine from Honolulu County (21).

## Discussion

Feral swine brucellosis seropositivity rates in Hawai‘i were previously unknown. They were presumed comparable to the rates seen in feral swine from the conterminous US, at ~10% (44, 45). Analysis of 15 years of feral swine serum samples from Hawai‘i in the present study found rates were at least double those seen on the mainland US (~10 vs. ~23%). Feral swine sampled from Kaua‘i and Moloka‘i were negative for brucellosis on both BAPA and FPA tests though caution is recommended when interpreting this result due to small sample sizes on these islands. Seropositivity rates were highest on the island of Maui even though limited watershed sampling occurred on the island. The Waiakoa watershed had a smoothed seropositivity rate of 90.11% followed by a 56.91% seropositivity rate in the Kailua Gulch watershed. These rates on Maui were higher than any other watersheds sampled in this study. A 2023 outbreak of brucellosis in a mid-size domesticated swine operation occurred in the Waiakoa watershed and could be the result of feral swine spillover (46).

LISA analysis identified clusters of high seroprevalence watersheds on O‘ahu and the island of Hawai‘i. The positivity rate was ~35% across this cluster on the north shore of O‘ahu which is higher than the 15-year average of 23.77% found in the sampled study population. The second cluster in the central and western portion of the island had seropositivity rates near the average. These two clusters are second order geographic neighbors and feral swine could feasibly move between the two clusters. These areas of the island are high in agricultural land use compared to others. Another possible effect on seropositivity is that swine tend to move in family groups and animals with close physical contact are more likely to infect one another. Due to the length of the study, this effect should be sufficiently diminished. The high seroprevalence watersheds on the island of Hawai‘i cluster between Mauna Kea and Mauna Loa, an area that has increased levels of agriculture compared to other parts of the island and had seropositivity rates at or above the state average. Some watersheds could have higher seropositivity due to the number of feral swine in those areas. More accurate counts could help with determining their population levels. It could also be due to the different types of swine in those areas. Two swine breeds were introduced to the islands. The Polynesian swine and, hundreds of years later, the Eurasian swine. It is unknown whether the current distribution of animals is correlated with these historical populations or if it plays a role in disease prevalence. It is also worth noting that a previous study found that 52% of *B*. suis culture positive swine from across the USA were positive by brucellosis FPA (21); indicating a potential underestimation of *B. suis* in Hawai‘i's feral swine as determined in this study.

Molecular epidemiological analysis suggests a genetic link between a *B. suis* strain in Tahiti and B00-0468 isolated from a cow in Hawai‘i in 2000, but not to *B. suis* in other parts of Polynesia. The other two *B. suis* strains isolated in Hawai‘i from cattle in 1993 and 2011 were more closely related to Eurasian and central American strains or mainland US strains, respectively. Whole-genome sequencing data is not as complete as the historical MLVA databases. Using available whole genome sequences, *B. suis* infecting domestic cattle in Hawai‘i as far back as 1993 and as recently as 2011 had similar genome sequences that were related to *B. suis* from the US mainland. Based on available MLVA and whole genome sequence analysis, a westward transportation from Eurasia or the Americas to Hawai‘i that established the biovar 1 *B. suis* in the central Pacific rather than an eastward movement from Asia or other parts of Polynesia can be speculated. A caveat is that the genomic information is rather limited. Other strains profiles have been published using out of date typing schemes incompatible with the MLVA analysis. *B. suis* biovar 3 has been reported in feral swine in Hawai‘i (21) however current typing and genomic data were not generated. This indicates a need for further sampling and genomic analysis to understand the molecular epidemiology of *B. suis* in the Hawaiian Islands.

Modern animal movement practices have reduced the introduction of zoonotic disease into Hawai‘i. Previous introduction through the mid-20th century is likely and could even have occurred during discovery of Hawai‘i by ancient Polynesian mariners. While the historical source of the zoonotic pathogen in Hawai‘i may be debatable, the presence of *B. suis* in terrestrial wildlife in Hawai‘i is not. The rate of brucellosis seropositivity in feral swine is double the national average of the US over the last 15 years and in some areas of the Hawai‘i it may be difficult to find feral swine that are negative for exposure. Feral pig hunting is practiced in Hawai‘i by using hunting dogs to track and capture the animals, a situation where both human hunters and their dogs could be exposed. A limited study found 1/7(14%) of tested hunting dogs on O‘ahu and 2/49 (4%) on Hawai‘i had titers indicating *Brucella* exposure (47). Future work focused on disease exposure or infection in hunting dogs and hunters could help understand risk to those groups. The three cases of domestic cattle infected with *B. suis* nearly 20 years apart, and recent domestic swine outbreaks of brucellosis indicate a pernicious threat to agriculture in the state. As an isolated island chain, Hawai‘i supports agriculture that provides locally sourced foods to residents. Difficult to control feral swine populations that act as reservoirs for brucellosis and transmit disease to many different species, including livestock and humans, threaten the public health and the state's burgeoning agricultural goals.

Identification of high positivity watersheds and hotspot clusters can be used by policy makers and stakeholders to focus limited resources for the most impact. Eliminating feral swine in areas of the highest zoonotic burden can reduce brucellosis infection risk to humans and livestock. This study has laid the groundwork for future studies into brucellosis in the state and in other areas of Polynesia. More genomic data and animal movement information are needed to understand the dynamics of brucellosis among terrestrial wildlife in the Hawaiian Islands and transmission to humans. In the future, occurrence of *Yersinia enterolitica* infections in swine should also be investigated, as swine with high antibody levels to *Y. enterolitica* can cross-react to brucella serological tests and yersiniosis is a zoonoses that impacts public health in Hawai‘i. However, the spike in positivity on Maui coincident with a known brucella outbreak and the spillover of *B. suis* into cattle and humans support the notion of higher levels of circulating brucellosis in the feral swine population. The increased specificity of the FPA test over other brucellosis test like Rose-Bengal Test (RBT) and the complement fixation test (CFT) support our serological findings. Additionally, the delicate island ecosystems are inextricably linked and disease on land can be transmitted to fragile marine ecosystems (48, 49). Brucellosis in marine mammals is recognized as an emerging infectious disease. Since the first described marine mammal brucellosis infection in 1994 (50), there have been several strandings of marine mammals infected with *Brucella* in Hawai‘i (51, 52) and evidence of exposures in other marine animals (53). Understanding brucellosis across terrestrial and marine environments can provide valuable insight to protecting agricultural, wildlife, and public health in Hawai‘i, the Pacific basin, and the broader Asia-Pacific region.

## Data Availability

The original contributions presented in the study are included in the article/Supplementary material, further inquiries can be directed to the corresponding author.

## Supplementary material

Supplementary Figure S1 Feral swine serum sampling numbers across Hawai'i watersheds in this study. A map of the major islands of Hawai'i displaying the 558 watershed divisions. The watersheds are colored according to the number of feral swine sampled in that watershed during the course of the study. Watersheds that are white did not have any feral swine sampled.
