# Supplementary figures and images for: Tracking sero-molecular trends of swine brucellosis in Hawai‘i and the central Pacific
Source: Front Public Health. 2024 Sep 4;12:1440933. doi: 10.3389/fpubh.2024.1440933 (PMC11408231; doi:10.3389/fpubh.2024.1440933)

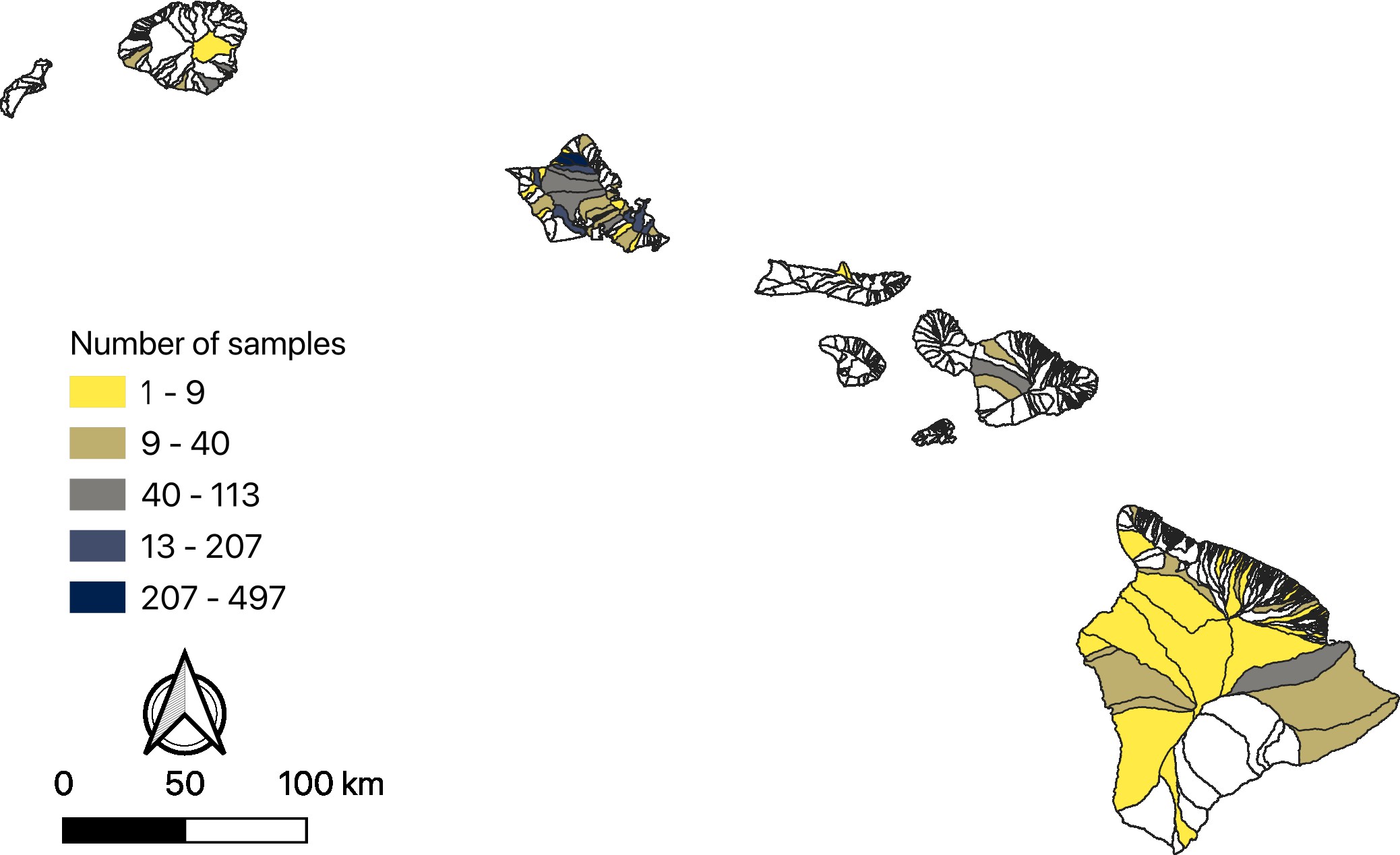

Supplement: Supplementary Figure S1 — Feral swine serum sampling numbers across Hawai'i watersheds in this study. A map of the major islands of Hawai'i displaying the 558 watershed divisions. The watersheds are colored according to the number of feral swine sampled in that watershed during the course of the study. Watersheds that are white did not have any feral swine sampled. [file Image_1.JPEG]
